# Lnc-MAP6-1:3 knockdown inhibits osteosarcoma progression by modulating Bax/Bcl-2 and Wnt/β-catenin pathways

**DOI:** 10.7150/ijms.47405

**Published:** 2020-08-21

**Authors:** Hao Lin, Tingrui Wu, Lijiao Peng, Wenmei Su, Yingxin Wang, Xiao Li, Qianzheng Liu, Chanli Zhong, Jing Huang, Bo Wei

**Affiliations:** 1 Department of Orthopedics, Affiliated Hospital of Guangdong Medical University, Zhanjiang, Guangdong, 524001, China.; 2 Oncology Center, Affiliated Hospital of Guangdong Medical University, Zhanjiang, Guangdong, 524001, China.; 3 Institute of Laboratory Medicine, Guangdong Medical University, Dongguan, Guangdong, 523808, China.

**Keywords:** Lnc-MAP6-1:3, Osteosarcoma, Oncogenic lncRNA, Bax/Bcl-2, Wnt/β-catenin

## Abstract

Osteosarcoma (OS) is the most common type of malignant bone tumor that affects children and adolescents. Still, the cellular and molecular mechanisms driving the development of this disease remain poorly understood. In this study, numerous dysregulated lncRNAs were identified by RNA-seq. As a result, we were able to find a novel lncRNA Lnc-MAP6-1:3 which is highly expressed in osteosarcoma. Using a set of approaches including gene knockdown, RT-PCR, oncogenic function assay and western blotting, we observed that knockdown of Lnc-MAP6-1:3 expression suppressed cell proliferation and colony formation, and promoted apoptosis *in vitro*. For the first time, we have identified that Lnc-MAP6-1:3 potentially influence the malignant behavior of osteosarcoma via Bax/Bcl-2 and Wnt/β-catenin signaling pathways. Henceforth, Lnc-MAP6-1:3 may provide a new molecular route of research and therapeutic applications for the diagnosis and treatment of osteosarcoma.

## Introduction

Osteosarcoma (OS) is the most common type of bone cancer. Frequent OS sites are metaphysis of long bones in children and adolescents 1. The characteristics of rapid growth and early metastasis are the main factors for the poor prognosis of osteosarcoma 2, 3. Due to the increased five-year disease-free survival, the combination of limb salvage and neoadjuvant chemotherapy has become a standard treatment strategy for osteosarcoma patients.

Due to the aggressive behavior of OS, ~20% of patients are affected by pulmonary metastasis at the time of initial diagnosis, and another 30% of patients may develop pulmonary metastasis within 2-3 years after diagnosis 4. Therefore, it is of seminal importance to define the mechanisms and underlying effectors which drive the malignancy of this disease.

It has been perceived that more than 90% of the human genome is actively transcribed, but less than 2% of the transcripts encode proteins, while the vast majority are defined as non-coding RNAs (ncRNAs), including miRNA, circRNA, and long noncoding RNA (lncRNA) 5, 6. LncRNAs, a diverse class of largely functional transcripts with a length of more than 200 nucleotides, have been involved in various cellular processes, such as oncogenes or tumor suppressor genes, cell proliferation, apoptosis, differentiation, invasion, metastasis, and disease pathogenesis 7-10. A number of evidences have also indicated that differentially expressed lncRNAs can be associated with tumorigenesis in mostly all cancer types, where they may perform important regulatory functions. In fact, lncRNAs have been involved in various malignancies, such as esophageal 11, 12, lung 13, 14, breast 15, 16, gastric 17, 18 and liver 19, 20 cancers. Although a growing number of lncRNAs have been characterized, the exact function of lncRNAs in OS remains poorly understood.

Currently, studies focusing on lncRNA-mediated regulation of osteosarcoma have mainly centered on lncRNAs previously described in other malignant tumors, such as MALAT1 21, HOTAIR 22, H19 23, TUG1 24 and MEG3 25. Research on lncRNAs that specifically relate to osteosarcoma has been poorly performed. Based on RNA sequencing data, here we report a novel lncRNA, called Lnc-MAP6-1:3, which is highly expressed in osteosarcoma cells. Knockdown of Lnc-MAP6-1:3 is capable of decreasing cell proliferation, invasion and migration, as well as increasing apoptosis. Concomitantly, the expression of key proteins in Bax/Bcl-2 and Wnt/β-catenin signaling pathway appear to be down-regulated in OS cells. Thus, Lnc-MAP6-1:3 may regulate the development of osteosarcoma through Bax/Bcl-2 and Wnt/β-catenin signaling pathway.

## Materials and Methods

### Cell culture

The human cell lines hFOB1.19, U2OS, MG63 and HOS were purchased from the Chinese Academy of Sciences Cell Bank. hFOB1.19 cells were grown in D-MEM/F-12 medium (Gibco, Carlsbad, CA, USA) supplemented with 10% fetal bovine serum (FBS) (Gibco, Sydney, Australia) and maintained at 33.5 °C with a humidified atmosphere of 5% CO_2_. U2OS cells were grown in RPMI-1640 medium (Gibco, Carlsbad, CA, USA) supplemented with 10% FBS and 1% penicillin/streptomycin (Gibco, Grand Island, NY, USA), and incubated at 37°C in a humidified atmosphere of 5% CO_2_. MG63 and HOS cells were grown in MEM medium (Gibco, Carlsbad, CA, USA) supplemented with 10% FBS and 1% penicillin/streptomycin, and incubated at 37°C in a humidified atmosphere of 5% CO_2_.

### RNA sequencing assay

Total RNA from tissues (or cells) was isolated using Hipure Total RNA Mini Kit (Magen) according to the protocol. The concentration and integrity of the extracted total RNA was estimated by Qubit 3.0 Fluorometer (Invitrogen, Carlsbad, California), and Agilent 2100 Bioanalyzer (Applied Biosystems, Carlsbad, CA), respectively.

RNA-seq library was prepared with approximately 1μg of total RNA using KAPA RNA HyperPrep Kit with RiboErase (HMR) for Illumina® (Kapa Biosystems, Inc., Woburn, MA). Briefly, ribosomal RNA was removed from the total RNA. Next, the ribominus RNAs were fragmented and then first strand and directional second strand synthesis were performed. Then the A tailing and adapter ligation were performed with the purified cDNA. Finally, the purified, adapter-ligated DNA was amplified. The library quality and concentration were assessed by utilizing a DNA 1000 chip on an Agilent 2100 Bioanalyzer. Accurate quantification for sequencing applications was determined using the qPCR-based KAPA Biosystems Library Quantification kit (Kapa Biosystems, Inc., Woburn, MA). Each library was diluted to a final concentration of 10 nM and pooled equimolar prior to clustering.150bp paired-end (PE150) sequencing was performed on all samples. The differential expression of lncRNAs and mRNAs were annotated according to fold change/*P* value/FDR filtering (fold change ≥ 1.5, *P* value < 0.05, and FDR < 0.05).

### Functional enrichment analysis

The functions and pathway enrichment of differentially expressed genes were analyzed using Kyoto Encyclopedia of Genes and Genomes (http://www.genome.jp/kegg/). This repository contains information on how molecules and genes are networked, and it was also used for pathway mapping.

### Patients and controls

A total of 6 patients with primary OS, treated at the Department of Orthopedics, Affiliated Hospital of Guangdong Medical University, were enrolled based on a confirmed histological diagnosis. None of the patients received radiotherapy and/or chemotherapy before surgery. OS tissues and their matched adjacent normal counterparts were obtained from patients who underwent complete resection surgery. All tissue samples were immediately frozen after extraction in liquid nitrogen. This research was approved by the Review Board and Ethics Committee of Affiliated Hospital of Guangdong Medical University. Informed consents were provided by all enrolled patients.

### RNA isolation and qRT-PCR

Total RNA was isolated from tissues or cultured cells using Trizol reagent (Invitrogen, Carlsbad, CA, USA) according to the manufacturer's instructions. Extracted RNA was spectrophometrically quantified using a NanoDrop equipment (Thermo Fisher Scientific, Waltham, MA, USA). First strand cDNA was generated using a cDNA Synthesis kit (Takara, Otsu, Japan). Real-time PCR was performed using TB Green PCR Master Mix (Takara) with ABI StepOne Real-Time PCR System (Applied Biosystems 7500, Foster City, CA, USA). The expression levels of respective lncRNAs were normalized according to GAPDH expression, by using an optimized comparative Ct (2^-ΔΔCt^) value method. The real-time PCR reactions were performed in triplicates. The primers used were as follows: Lnc-MAP6-1:3 (forward, 5′-ACAACGCCAGACACGATGCTTC-3′ and reverse, 5′-GCAGTGAGGCGGATTGAGAAGG-3′); and GAPDH (forward, 5′-CACCCACTCCTCCACCTTTG-3′ and reverse, 5′-CCACCACCCTGTTGCTGTAG-3′).

### SiRNA and transfection

Respective siRNAs were synthesized by GenePharma (Shanghai GenePharma Co., Ltd., Shanghai, China). The following siRNA sequences were used: Sense: 5′-GCUCCUUCUCAAUCCGCCUTT-3′, Antisense: 5′-AGGCGGAUUGAGAAGGAGCTT-3′; siNC: Sense 5′-UUCUCCGAACGUGUCACGUTT-3′, Antisense 5′-ACGUGACACGUUCGGAGAATT-3′. A total of 3×10^5^ OS cells were seeded per well in a 6-well plate, and transiently transfected with 10 nM siRNA using Lipofectamine iMAX kit (Invitrogen), according to the manufacturer's instructions. OS cells were harvested for RNA and protein extraction after 48-72 hours of incubation with respective siRNAs.

### Cell proliferation assay

The cell proliferation status was detected according to manufacturer's instructions using Cell Counting Kit (CCK)-8 (Dojindo Laboratories, Kumamoto, Japan). About 1×10^3^ OS cells were plated in 96-well plates and, 72 h after siRNA transfection, CCK-8 solution was added at 10 μl/well (during the last 1 h of cell culture). The optical density (OD) values were measured at 450 nm. All experiments were performed in triplicates.

### Colony formation assay

Upon siRNA transfection, two hundred OS cells were plated into each well of 6-well plates. Transfected cells were incubated in RPMI-1640 or MEM medium with 10% FBS at 37 °C. After culturing for 14 days, cells were fixed with 20 % methanol and stained with 0.1% crystal violet. Colonies defined as greater than 50 cells were counted.

### Apoptosis detection

Cell apoptosis was evaluated using Annexin V fluorescein isothiocyanate and propidium iodide (Annexin V-FITC/PI) apoptosis detection kits (BD Biosciences, Franklin Lakes, NJ, USA). Briefly, 3×10^5^ cells/well were plated into 6-well plates for 24 hours, and then transfected with 10 nM siRNA for further 48 hours. Cells were then collected, washed twice with PBS, and stained with Annexin V-FITC and PI according to the manufacturer's protocols. Samples were analyzed by flow cytometry. Both early and late apoptotic cells were recorded as apoptotic cells, and the results were expressed as a percentage from the total number of cells.

### Cell migration and invasion assays

OS cell lines were harvested and collected 48 h after siRNA transfection. For the migration assays, 2.5 × 10^4^ cells in 100 μl medium with 1% FBS were seeded on the upper chambers (Costar Inc., USA). For the invasion assays, the upper chambers were pre-coated with 250 μg/ml Matrigel (Corning), and then incubated at 37°C for 4 h, followed by the placement of 2.5x10^4^ transfected cells into the upper chambers. To allow cell migration, the lower chambers of the transwells were filled with 750 μl medium containing 20% FBS. After the cells were incubated for 24-48 h, the cells remaining on the upper membrane were removed by scrubbing with a cotton swab. The cells that passed through the membrane were then fixed in 90% ethanol and stained with crystal violet solution. Five random fields per chamber were counted, using an inverted microscope (Olympus, Tokyo, Japan).

### Western blotting

OS cells were harvested 72 h after transfection and lysed with RIPA buffer (Solarbio, Beijing, China). The samples were submitted to SDS-PAGE (Solarbio, Beijing, China) at 80 V for 3 h, and then transferred to PVDF (Millipore, Billerica, MA) membranes for another 3 h. After the membranes were blocked in 5% skim milk at room temperature for 1 h, they were incubated with primary antibodies overnight at 4 °C. Membranes were further probed with secondary antibodies at room temperature for 1 h and the immunoreactive bands were detected by Chemiluminescent and/or Fluorescent Imaging System (Tanon 5200, Shanghai, China). Rabbit monoclonal antibodies against GAPDH, Bax, Bcl-2, β-catenin, TEF1, c-Myc, cyclin D1, MMP-7 [Cell Signaling Technology (CST), Inc., Danvers, MA, USA] were used at 1:1,000 dilution. The secondary antibodies (goat anti-rabbit IgG/HRP) were used at 1:2,000 dilution [Cell Signaling Technology (CST), Inc., Danvers, MA, USA]. The band intensity of the target proteins was normalized according to the intensity of GAPDH band.

### Statistical analysis

Measurement data are presented as mean ± standard deviation (SD), and were evaluated by unpaired Student's *t*-test. All data are continuous variables and follow a normal distribution. Statistical analysis and graph representations were performed using GraphPad Prism 7 Software (GraphPad, San Diego, CA). Statistical significance was noted at *P* < 0.05. Three independent experiments were performed for cell-based assays, unless otherwise stated.

## Results

### Profile of lncRNA expression in OS

**Figure 1:**
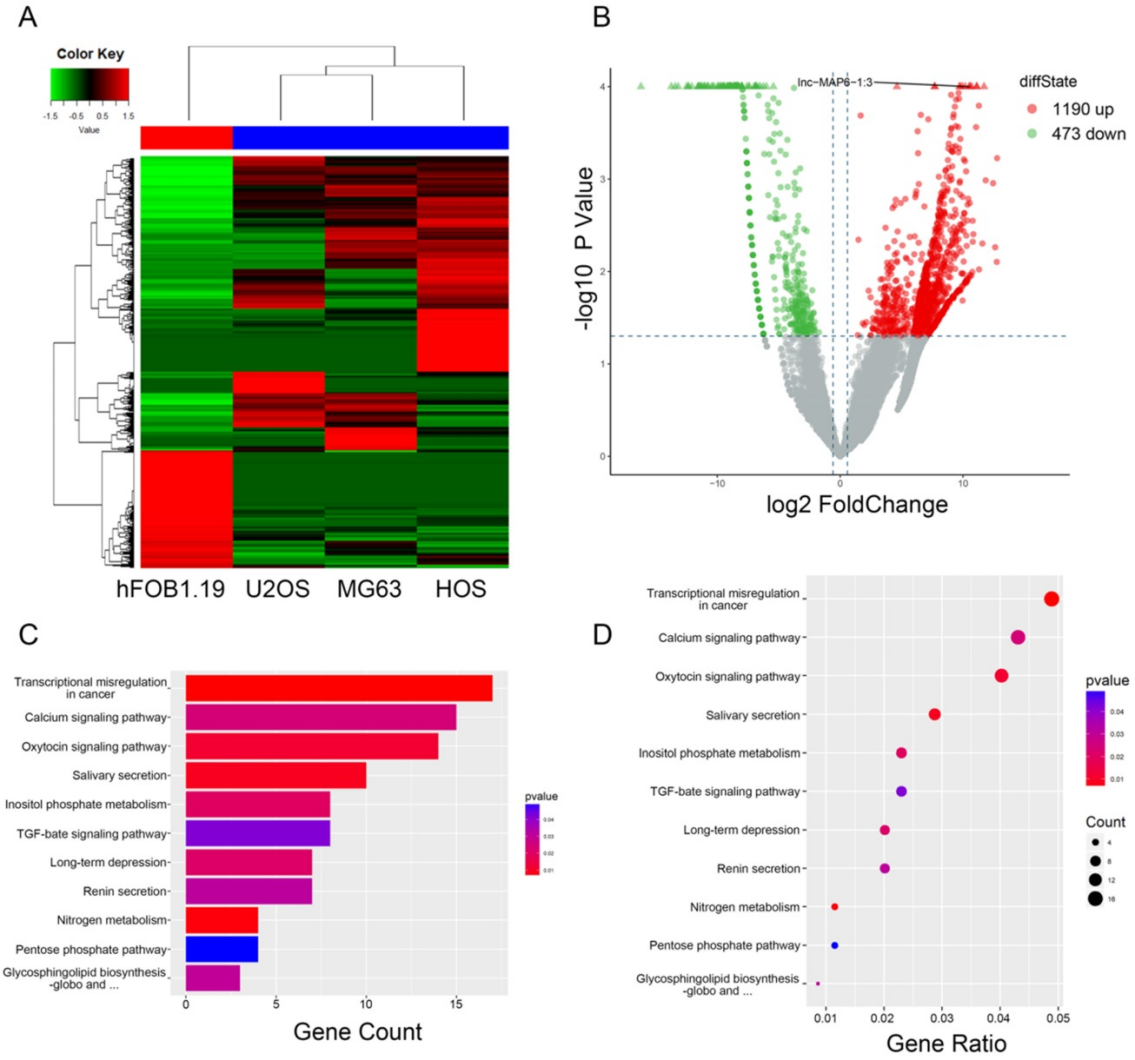
Gene expression profile differences between the OS cell lines and the osteoblasts, and enrichment analysis of KEGG pathways for differentially expressed lncRNA/mRNAs. (**A**) Hierarchical clustering indicates lncRNA profiles. Red and green indicate high and low expression, respectively. In the heat map, the columns represent samples, and the rows represent respective lncRNAs. (**B**)Volcano plots were used to distinguish the differentially expressed lncRNAs. The vertical lines correspond to 1.5-fold difference and the horizontal line represents a *P* value of 0.05. (**C and D**) Pathway analysis based on the KEGG database.

To initially investigate lncRNA expression in osteosarcoma, total RNA of three osteosarcoma and one osteoblast cell lines were analyzed by RNA-sequencing. After data screening (fold change ≥ 1.5, *P* value < 0.05), lncRNA expression profiling suggested the presence of 1,663 differentially expressed lncRNAs, including a total of 1,190 upregulated and 473 downregulated lncRNAs (Figure 1B). The hierarchical clustering of the differentially expressed lncRNAs is listed (Figure 1A). KEGG pathway enrichment analysis of the differentially expressed lncRNA/mRNAs demonstrated their association with 11 distinct signaling pathways. “Transcriptional misregulation in cancer pathway” was one of the most significantly involved pathways (Figure 1C-D).

### Lnc-MAP6-1:3 expression was increased in OS tissues and cell lines

**Figure 2:**
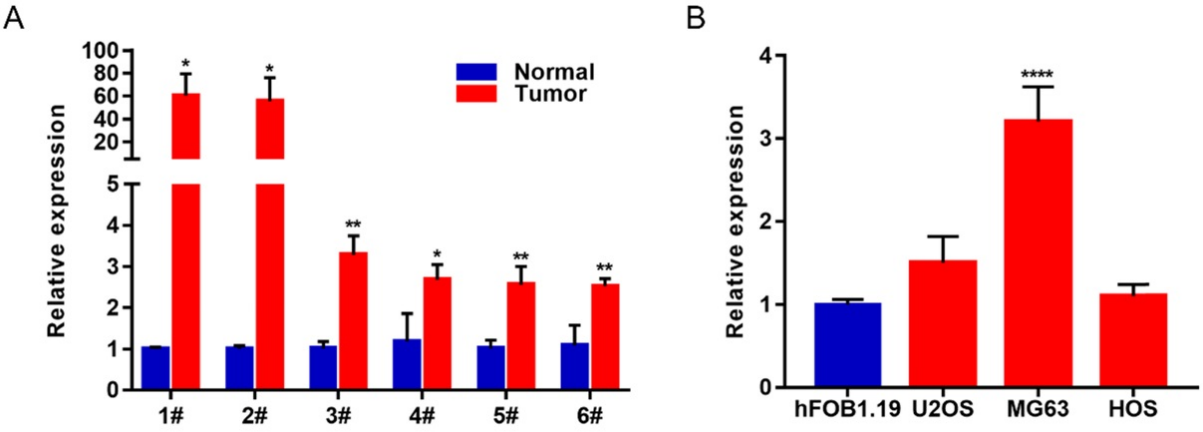
Lnc-MAP6-1:3 expression was increased in OS tissues and cell lines. (A) Lnc-MAP6-1:3 expression was higher in OS tissues by RT-PCR, including 6 OS tissues and 6 corresponding adjacent OS tissues. (**B**) Lnc-MAP6-1:3 was increased in OS cell lines as compared to the osteoblasts. Data shown are mean ± SD (n = 3) (*P < 0.05, **P < 0.01, ****P < 0.0001).

In order to study the biological function of lncRNAs in osteosarcoma, we selected the most differentially expressed Lnc-MAP6-1:3 from the list of upregulated lncRNAs (fold change ≥ 1.5, *P* value < 0.05, and FDR < 0.05) identified in this study. To verify the reliability of the RNA-Seq result, 6 paired OS tissues and their corresponding adjacent tissues were collected to further validate Lnc-MAP6-1:3 as an upregulated lncRNA by qRT-PCR. In consonance with the RNA-Seq results, Lnc-MAP6-1:3 was markedly increased in OS tissues when compared with adjacent non-tumor tissues (Figure 2A). Moreover, qRT-PCR assays also indicated that Lnc-MAP6-1:3 was upregulated in OS cell lines (Figure 2B).

### Knockdown of Lnc-MAP6-1:3 expression in OS cells suppresses cell proliferation and colony formation *in vitro*

**Figure 3:**
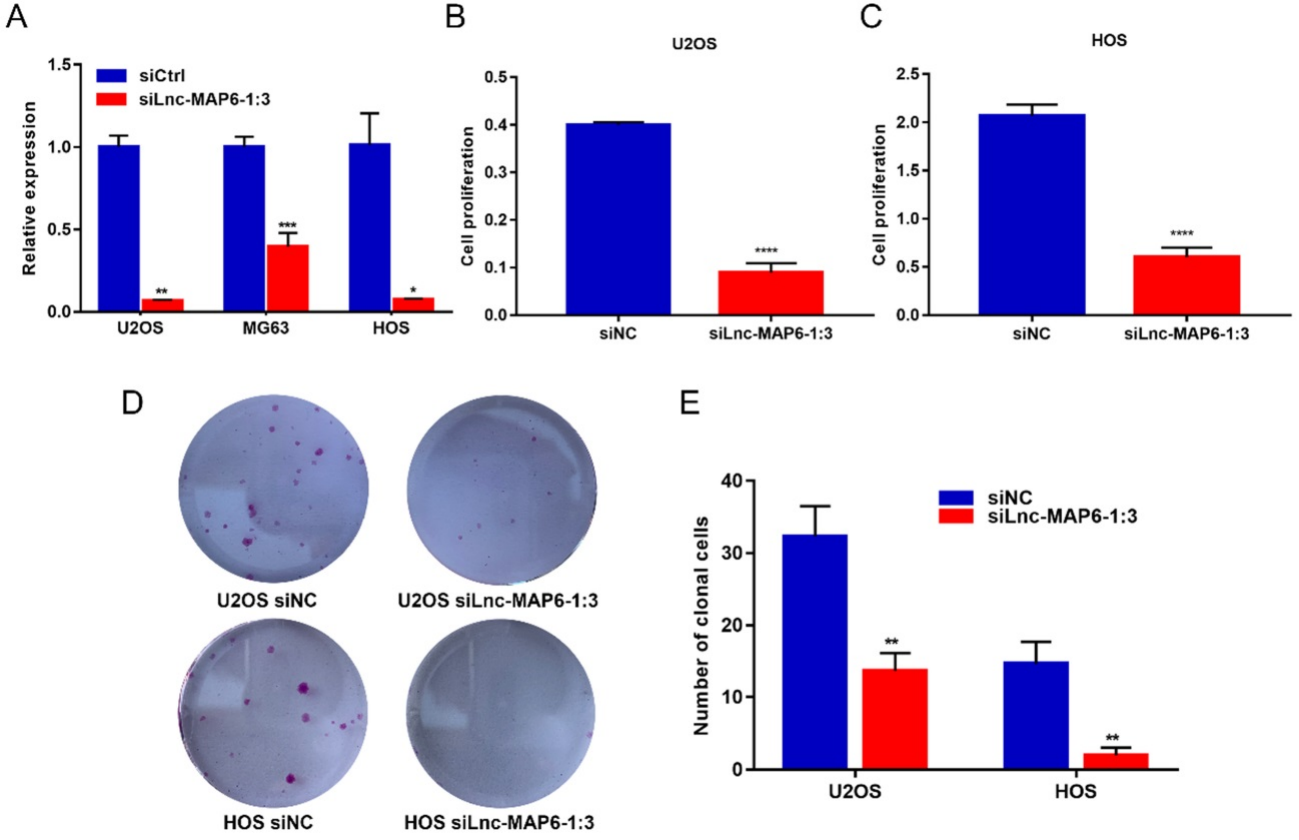
Effects of knockdown of Lnc-MAP6-1:3 on OS cells viability. (**A**) The Lnc-MAP6-1:3 expression level indicates the knockdown efficiency of siRNA, determined by qRT-PCR in 3 OS cells transfected with siLnc-MAP6-1:3. (**B-C**) CCK-8 assays were used to determine the cell viability after Lnc-MAP6-1:3 knockdown, by siRNA transfection in U2OS and HOS cells. (**D-E**) Colony formation in U2OS and HOS cells after Lnc-MAP6-1:3 knockdown. Bar chart illustrates the number of counted colonies. Data shown are mean ± SD (n = 3) (*P < 0.05, **P < 0.01, ***P < 0.001, ****P < 0.0001).

To elucidate the function of Lnc-MAP6-1:3 *in vitro*, we first transfected U2OS, MG63 and HOS cell lines with siRNA to knockdown Lnc-MAP6-1:3 expression. qRT-PCR assays indicated that the siRNA transfection provided a strong suppression of Lnc-MAP6-1:3 (> 90% inhibitory rate) in U2OS and HOS and, therefore, these two cell lines were selected for further analyses (Figure 3A). Subsequently, we found that cell proliferation (as measured by CCK-8 assays) was significantly decreased upon Lnc-MAP6-1:3 knockdown in U2OS and HOS cells (Figure 3B-C). Consistent with these observations, knockdown of Lnc-MAP6-1:3 expression significantly inhibited the ability of colony formation of OS cells when compared with a negative control (Figure 3D-E). These results suggest that Lnc-MAP6-1:3 may play an oncogenic role in regulating OS cell growth.

### Knockdown of Lnc-MAP6-1:3 promoted apoptosis in OS cells

**Figure 4:**
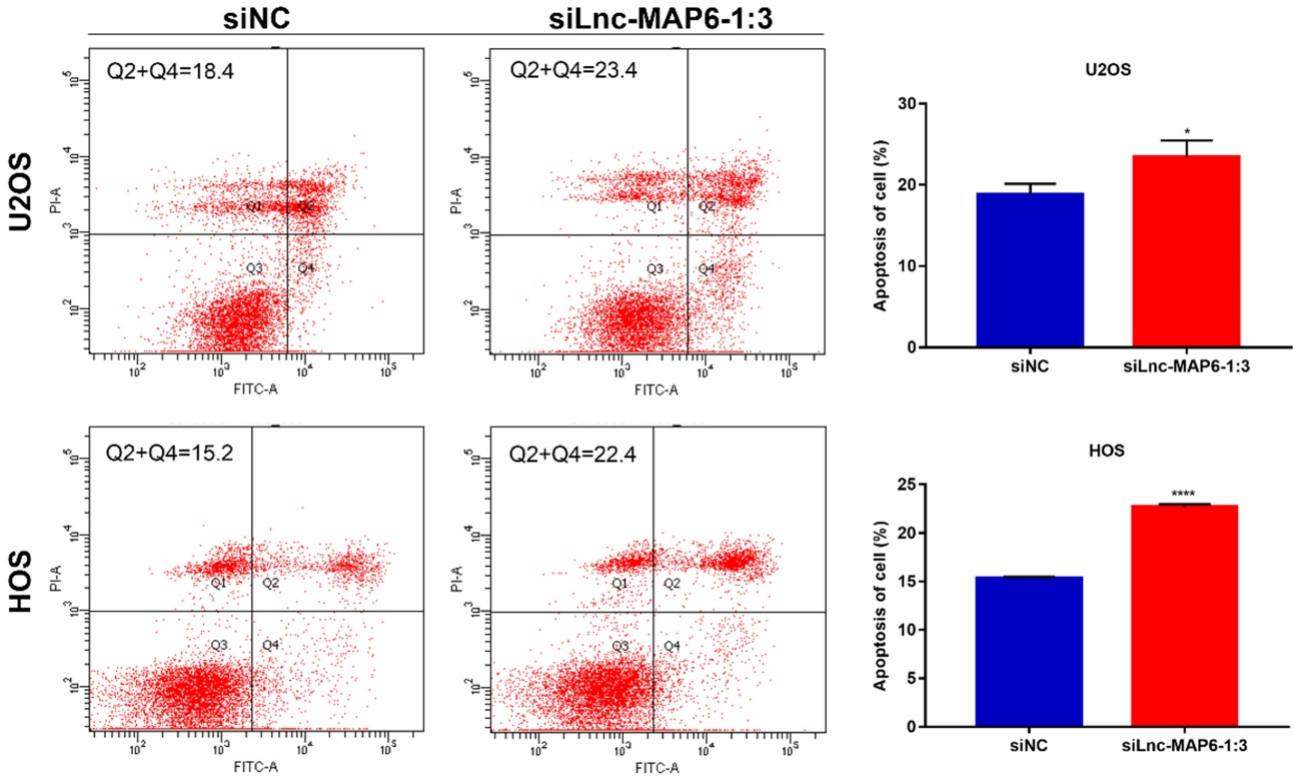
Effects of Lnc-MAP6-1:3 on the apoptosis of OS cells. U2OS and HOS cells were treated with siLnc-MAP6-1:3 for 48 hrs. Apoptosis was further detected using annexin V-FITC/PI dual staining by flow cytometry. The bar chart illustrates the counting number of early and late apoptotic cells. Data shown are mean ± SD (n = 3) (*P < 0.05, ****P < 0.0001).

Apoptosis is known to play an important part in tumor progression. Therefore, flow cytometry was used to analyze a potential anti-apoptotic role of Lnc-MAP6-1:3 in OS cells. Flow cytometry analysis indicated that apoptosis was increased after Lnc-MAP6-1:3 knockdown in OS cells (Figure 4). These findings suggest that Lnc-MAP6-1:3 might be involved in the regulation of cell apoptosis in OS cells.

### Knockdown of Lnc-MAP6-1:3 expression in OS cells inhibits migration and invasion *in vitro*

**Figure 5:**
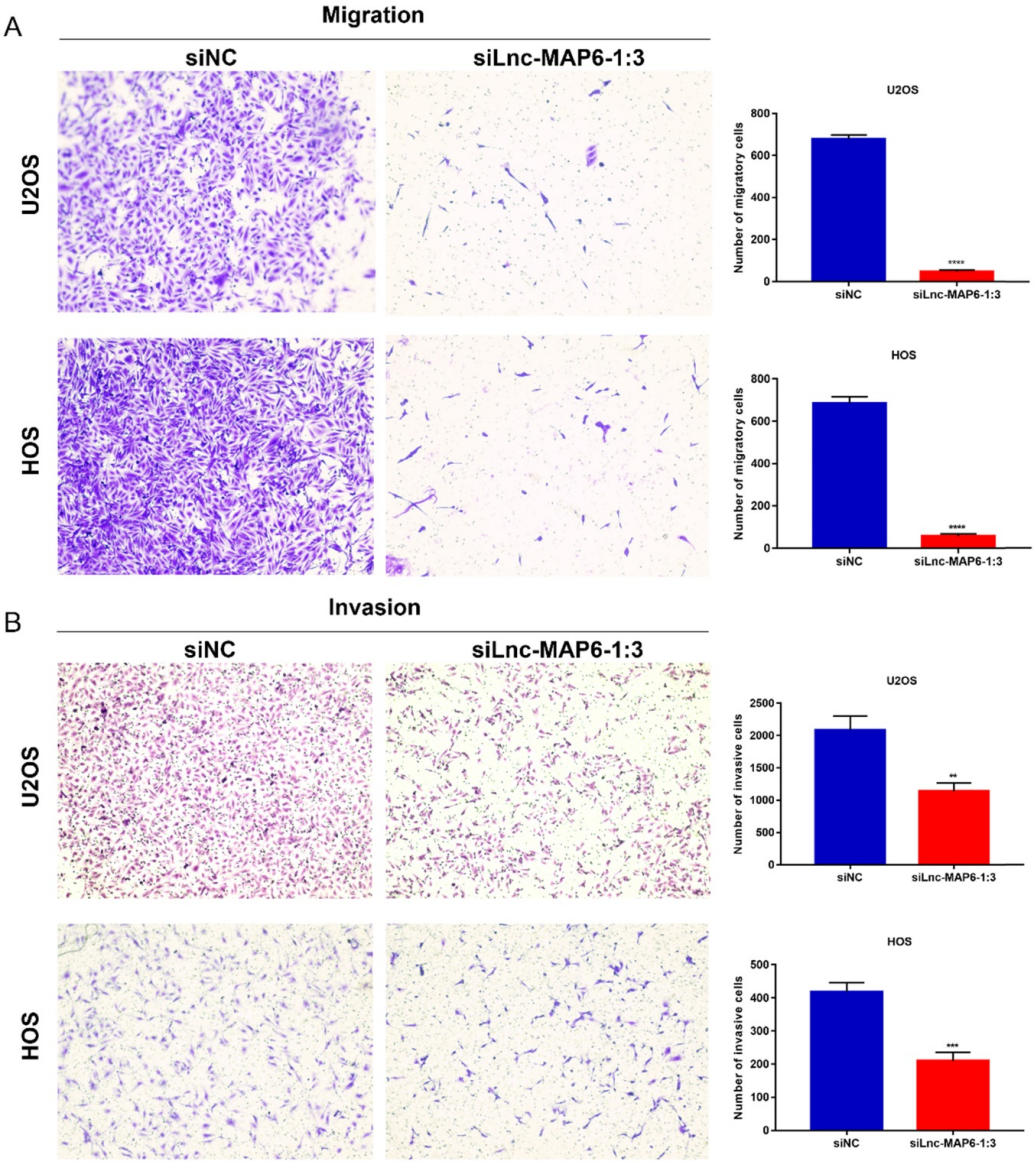
Knockdown of Lnc-MAP6-1:3 inhibits migration and invasion. Transwell migration (**A**) and invasion (**B**) were decreased after siLnc-MAP6-1:3 transfection in U2OS and HOS cells. The bar chart shows the number of cells that passed through the transwell chamber. Data shown are mean ± SD (n = 3) (**P < 0.01, ***P < 0.001, ****P < 0.0001).

Cell migration and invasion are important aspects of cancer metastasis, which may be related to the dissolution of cell matrix membrane and/or tumor cell metastasis to adjacent tissues. To evaluate a putative function of Lnc-MAP6-1:3 in the cancer cell migration and invasion, we performed transwell assays using OS cells. Downregulation of Lnc-MAP6-1:3 levels significantly decreased the number of migratory and invasive cells when compared with the controls (Figure 5A-B). These results imply that Lnc-MAP6-1:3 may be involved in mechanisms relevant to the metastatic potential of OS.

### Lnc-MAP6-1:3 knockdown up-regulates the expression of pro-apoptotic proteins and down-regulates anti-apoptotic factors

**Figure 6:**
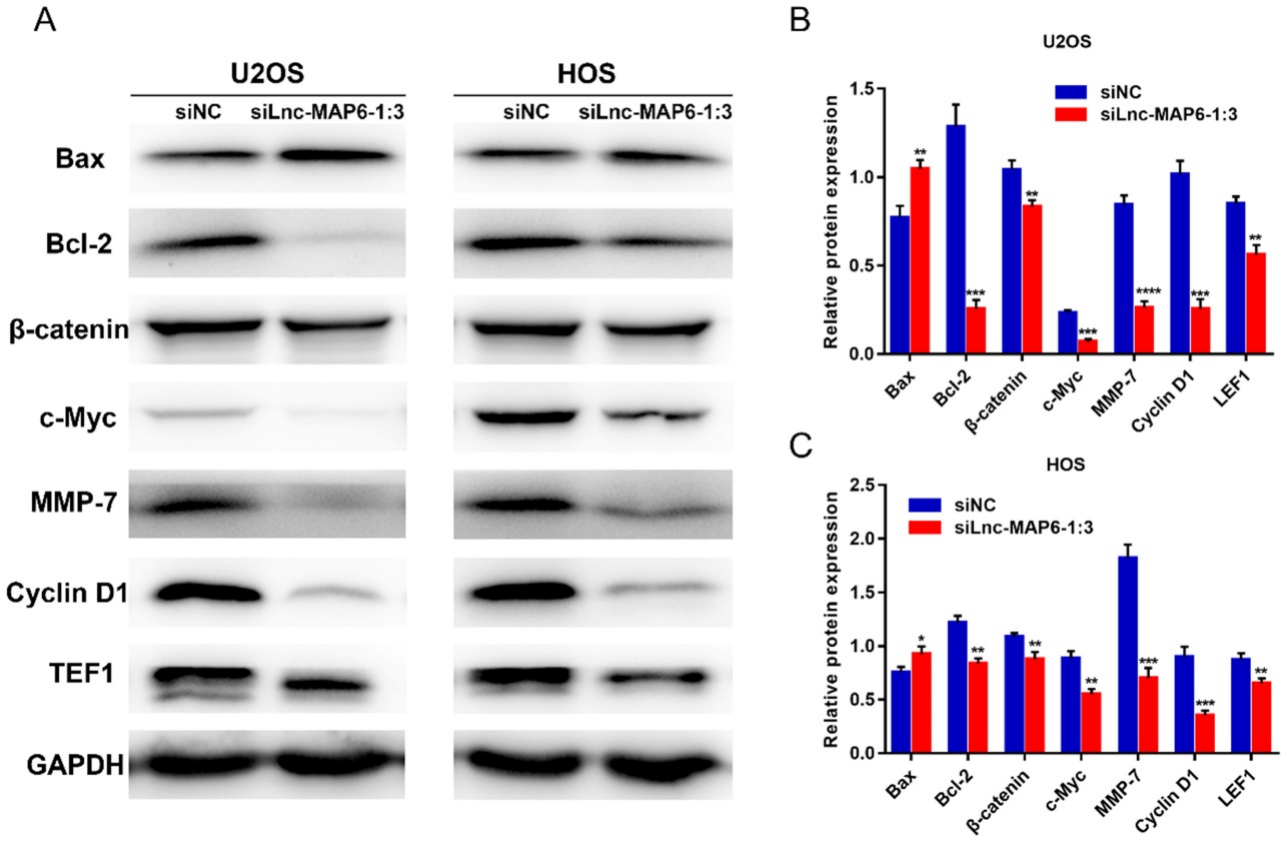
Target proteins regulated by Lnc-MAP6-1:3 knockdown. (**A**) Protein levels of Bcl-2, β-catenin, TEF1, c-Myc, cyclin D1 and MMP-7 were down-regulated by Lnc-MAP6-1:3 siRNA in U2OS and HOS cells, while the protein levels of Bax were up-regulated. GAPDH was used as a protein loading control. The bar chart shows respective protein levels of U2OS (**B**) and HOS (**C**) cells after normalization. Data shown are mean ± SD (n = 3) (*P < 0.05, **P < 0.01, ***P < 0.001, ****P < 0.0001).

The Bcl-2 family proteins are important regulators of apoptosis, including both pro-apoptotic protein Bax and anti-apoptotic protein Bcl-2. The ratio of Bax/Bcl-2 levels is known to determine cell fate in numerous models. To further study the potential mechanism by which Lnc-MAP6-1:3 may induce apoptosis in human osteosarcoma cells, the expression of Bax and Bcl-2 was examined by western blotting. As expected, Lnc-MAP6-1:3 knockdown was able to profoundly up-regulate Bax but down-regulate Bcl-2 in OS cells (Figure 6A-C).

### Lnc-MAP6-1:3 promotes OS progression via Wnt/β-catenin signaling pathway

To explore the molecular signaling of Lnc-MAP6-1:3 towards the proliferation, migration and invasion of OS cells, we examined the levels of several proteins potentially involved in the progression of OS cells, upon transient Lnc-MAP6-1:3 knockdown. Protein levels of TEF1, c-Myc, cyclin D1 and MMP-7 were decreased in the Lnc-MAP6-1:3 knockdown OS cells when compared with negative controls (Figure 6A-C). Since these cancer-related proteins are known targets of the Wnt/β-catenin signaling pathway, we further examined the β-catenin levels in osteosarcoma cells after Lnc-MAP6-1:3 knockdown. Consistent with the downstream target proteins, β-catenin was also down-regulated in the Lnc-MAP6-1:3 knockdown OS cells (Figure 6A-C). These results suggest that Lnc-MAP6-1:3 is likely to regulate the progression of osteosarcoma by Bax/Bcl-2 and Wnt/β-catenin signaling pathways.

## Discussion

We have presently identified numerous dysregulated lncRNAs by RNA-seq. However, the relationship between most of lncRNAs and osteosarcoma remains unclear. The oncogenic role of Lnc-MAP6-1:3 in OS has not been previously reported and, therefore, the function and mechanism of this lncRNA were investigated in this study. We were able to verify that Lnc-MAP6-1:3 is overexpressed in human osteosarcoma tissues and osteosarcoma cell lines. Knockdown of Lnc-MAP6-1:3 expression can suppress cell proliferation, colony formation and, at the same time, promote apoptosis. Our results suggest that Lnc-MAP6-1:3 may be involved in the rapid growth of OS. In addition, Lnc-MAP6-1:3 knockdown inhibits cell migration and invasion, suggesting that silencing of Lnc-MAP6-1:3 expression might be involved in the biological behavior of metastatic osteosarcoma.

Progression of OS involves complex molecular mechanisms and activation of distinct signaling pathways 26, 27. Previous studies have shown that the Wnt/β-catenin signaling cascade plays a key role in the pathogenesis and growth of human osteosarcoma 28-31. In this study, we have found that multiple members of the Wnt/β-catenin pathway, such as β-catenin and its downstream targets (TEF1, c-Myc, cyclin D1 and MMP-7) were decreased upon Lnc-MAP6-1:3 knockdown in OS cells. Moreover, Bax and Bcl-2 levels were up- and down-regulated, respectively, in OS cells. According to recent reports, upregulation of Bax/Bcl-2 ratio can inhibit proliferation in human osteosarcoma cells 32. c-Myc is a human oncogene which contributes to multiple hallmarks of cancer. As a transcriptional factor, early studies identified that c-Myc transcriptional targets are involved in many biological processes, such as metabolism, cell growth, cell cycle regulation, and apoptosis 33. Cyclin D1 serves as a central regulator for cell cycle progression, and aberrant expression of this protein is a significant contributor to tumorigenesis 34. Matrix metalloproteinases (MMPs) compose a family of transcription factors capable of regulating the tumor microenvironment, mainly by the degradation of the extracellular matrix. The expression and activation of MMPs appears to be in almost all cancer types, and particularly related to tumor metastasis 35. Here we show that the Lnc-MAP6-1:3 function in OS apparently depends on the regulation of Bax/Bcl-2 and Wnt/β-catenin signaling pathway. Although we have a preliminary understanding of the role of Lnc-MAP6-1:3 in OS, further studies are needed to better elucidate the function of downstream Lnc-MAP6-1:3 targets, including particular binding proteins.

In summary, our study shows that Lnc-MAP6-1:3 influences the proliferation, colony formation, apoptosis, as well as migration and invasion in OS, by potentially regulating Bax/Bcl-2 and Wnt/β-catenin signaling pathways. In the near future, Lnc-MAP6-1:3 might serve as a new biological route for OS studies, as well as a putative therapeutic target for the diagnosis and treatment of this disease.
